# Non-alcoholic Fatty Liver in Children and Adolescents in Saudi Arabia

**DOI:** 10.7759/cureus.46380

**Published:** 2023-10-02

**Authors:** Gadah Mujlli, Moeber Mahzari, Ibrahim Alslamah, Jamil Syed, Aamir Omair, Mahumoud Abulmeaty, Dara Aldisi

**Affiliations:** 1 Simulation and Skills Development Center, Princess Nourah Bint Abdulrahman University, Riyadh, SAU; 2 Department of Community Health, King Saud University, Riyadh, SAU; 3 College of Medicine, King Saud Bin Abdulaziz University for Health Sciences, Riyadh, SAU; 4 Department Endocrinology, Ministry of National Guard Health Affairs, Riyadh, SAU; 5 King Abdullah International Medical Research Center, King Saud Bin Abdulaziz University for Health Sciences, Riyadh, SAU; 6 Department of Pediatrics, King Abdullah Specialized Children Hospital, Riyadh, SAU; 7 Department of Medical Education, King Saud Bin Abdulaziz University for Health Sciences, Riyadh, SAU; 8 Department of Medical Physiology, Zagazig University, Zagazig, EGY

**Keywords:** saudi arabia, non-alcoholic steatohepatitis, pediatrics, fatty liver, non-alcoholic fatty liver

## Abstract

Objectives

This study aims to describe the disease parameters of children and adolescents diagnosed with non-alcoholic fatty liver disease (NAFLD) in Saudi Arabia. It also investigates the disease's progression and compares clinical, biochemical, and radiological parameters at baseline and follow-up of patients with NAFLD. This study was done between two groups of patients: obese and those of average body weight.

Methods

A retrospective cohort study was conducted between 2014 and 2018 through retrieved data from medical records. It included all children aged between six to 18 years diagnosed with NAFLD. Medical history was taken from each medical record, liver function test results, cholesterol, blood pressure readings, and body weight. Data have been restored from King Abdullah Specialist Children's Hospital (KASCH)​, Security Forces Hospital (SFH), and King Khalid University Hospital (KKUH)*.*

Results

A total of 116 subjects met the inclusion criteria; 65 (56%) were male, and 81 (70%) were obese. The majority of subjects (n=112) had mild NAFLD, with (71%) obese and (29%) non-obese, followed by moderate NAFLD with 50% among obese and non-obese (N=2), and non-alcoholic steatohepatitis (NASH) with 100% non-obese (N=2). Data showed that patients' proportion of obese to non-obese is 70% (N=81) to 30% (N=35), respectively.

Conclusion

NAFLD was found to affect obese children and adolescents more than non-obese, and male patients had a higher proportion of NAFLD than females. Also, obese patients had more advanced stages of NAFLD than non-obese patients. Finally, most subjects had been diagnosed with mild stage while a few had developed NASH.

## Introduction

Non-alcoholic fatty liver disease (NAFLD) is one of the world's most common chronic liver diseases [1]. NAFLD is defined by excess fat in the liver parenchyma [2]. NAFLD severity ranges from simple liver steatosis (fatty liver) to non-alcoholic steatohepatitis (NASH), which might progress to cirrhosis and end-stage liver disease [1]. Obesity has become one of the major health concerns worldwide, and a considerable increase in NAFLD prevalence and other metabolic implications have been observed [3, 4]. Due to the coexistence of central obesity, diverticulosis, dyslipidemia, and insulin resistance, NAFLD is now considered the hepatic manifestation of metabolic syndrome (MetS) [3,4].

NAFLD is a significant public health concern since it is one of the most common causes of liver disease in pediatric age groups [5,6,7]. Studies and data about NAFLD in the pediatric population are generally rare [8]. While the prevalence of NAFLD among pediatric age groups varies between studies, the data available indicates that it is more prevalent in the obese pediatric population [8]. A meta-analysis conducted in 2015 estimated that the prevalence of NAFLD in children aged 1-19 years was 7.6% (95% CI 5.5-10.3%), and it increased to 34.2% (95% CI 27.8-41.2%) in NAFLD studies conducted in pediatric obesity clinics [9].

The progression of the disease from NAFLD to non-alcoholic steatohepatitis (NASH), the advanced fibrosis stage of NAFLD, varies among pediatric patients based on their population [10]. Unlike adults, pediatric NASH has two types; type 1 is characterized by steatosis, ballooning degeneration, and perisinusoidal fibrosis [11]. Type 2 NASH is characterized by steatosis, portal inflammation, and portal fibrosis. Incidence of type 2 is higher than type 1 in pediatric patients diagnosed with NAFLD. In a study by Schwimmer et al., 17% of the 100 children diagnosed with NAFLD presented with type 1 NASH, while 51% presented with type 2 NASH [11]. In another study by Schwimmer et al., out of the 742 pediatric patients aged 2-19 years, approximately 25% had NASH [12]. The prevalence of NASH among pediatric patients with NAFLD ranged from 20% to 50% at the time of diagnosis [10].

Ultrasound is the most commonly used method of evaluating steatosis and fibrosis among NAFLD patients due to the availability of the imaging modality, low risk to the patient, and non-invasive [13-15]. MRI elastography has advantages over ultrasound as it provides a comprehensive examination and quantification of fat in the liver. It is possible to accurately measure fat and liver fibrosis to diagnose NAFLD in a non-invasive manner, thus reducing the need for invasive liver biopsy [15]. Healthcare practitioners need to consider various factors to develop prevention and treatment approaches for NAFLD. These factors may play a part in the development and progression of the disease; they extend from metabolic risk factors to genetic, social, and environmental factors [8]. Early, effective intervention is critical to prevent disease progression into adulthood. Nevertheless, it is not often achieved through lifestyle modification alone [16].

There is scarce data on the prevalence of NAFLD in the Saudi pediatric population. However, due to the high prevalence of obesity in Saudi Arabia, with a prevalence reaching 9.3% in Saudi pediatric age groups, it is predicted that the prevalence of NAFLD will be relatively high and will continue to increase [17-19]. NAFLD has been linked with metabolic syndrome (MetS) and its components, which are considered risk factors for the disease's development and progression [20]. Clinicians suspect NAFLD based on the association of fatty liver combined with risk factors, mainly obesity, and after excluding other causes of liver disease [13]. Therefore, this study aimed to describe the disease parameters of children and adolescents diagnosed with NAFLD in Saudi Arabia and investigate the disease's progression. In particular, to compare clinical, biochemical, and radiological parameters at baseline and follow-up of the patients with NAFLD who were obese versus those having normal body weight.

## Materials and methods

Study design and subjects

This study was a retrospective cohort study in which data was retrieved from the medical records between 2014 and 2019 for patients aged 6 to 18 years diagnosed with NAFLD, who were categorized as obese or with normal body weight. It was conducted in three tertiary care hospitals located in Riyadh, Saudi Arabia: King Abdullah Specialist Children's Hospital (KASCH)​, Security Forces Hospital (SFH), and King Khalid University Hospital (KKUH).

A consecutive sampling technique was followed, and all patients eligible for the study were included. The inclusion criteria for the subjects were male and female patients (age 6-18 years) with medical records in KASCH, SFH, or KKUH hospitals and who were diagnosed with definitive NAFLD either with radiological tests or liver histology. The exclusion criteria included having a history of parenteral nutrition, bariatric or hepatobiliary surgery, human immunodeficiency virus (HIV) infection, known metabolic disorder, renal dysfunction, and short bowel syndrome.

Ethical approval was obtained from each center's research ethics committee. The database of each hospital was searched to collect the study sample. The search used the following keywords: non-alcoholic fatty liver disease, steatosis, and steatohepatitis. Then, all subjects who met the inclusion and exclusion criteria were included.

Study Parameters

Gender, age, and medical history were taken from each medical record. The medical history included the patient's diagnosis and how they were diagnosed with NAFLD. Results of liver function tests were collected. The test included alanine transaminase (ALT), aspartate transaminase (AST), alkaline phosphatase (ALP), gamma-glutamyl transpeptidase (GGT), and bilirubin. Other relevant variables were recorded, such as prothrombin time and C-reactive protein (CRP). Due to the association between MetS and NAFLD, MetS variables such as total cholesterol, high-density lipoprotein (HDL)-cholesterol, triglycerides, blood pressure readings, and weight were collected. NAFLD diagnosis method was recorded for all subjects when available. The NAFLD stages were recorded as well at baseline and upon follow-up. Based on radiological and biochemical data and the physician's report, the stages were categorized into mild steatosis, moderate steatosis, and NASH.

Statistical analysis

The subjects were divided into two groups: obese patients with NAFLD and non-obese patients with NAFLD. The independent variable was the presence of obesity, while the patient's parameters were the dependent variables. Data were analyzed using the Statistical Package for Social Sciences version 21 (IBM Inc., Armonk, New York). Student's T-test was used to perform the statistical tests. A statistical test with a p-value of <0.05 was considered significant. NAFLD diagnosis methods and stages were shown as frequencies and percentages.

## Results

Demographic data 

This study included 116 subjects; 65 (56%) were male, with the majority, i.e., 98 (84%), being more than ten years or older. The subjects were divided into two groups: group I included 81 (70%) obese patients with NAFLD, and group 2 included 35 (30%) non-obese patients with NAFLD.

**Table 1:** Study participants' demographic data (N=116) NAFLD - non-alcoholic fatty liver disease

Variables	n (%)
Female gender	51 (44%)
Male	65 (56%)
Age groups < 10 years old	18 (16%)
≥10 years old	98 (84%)
NAFLD and obesity	81 (70%)
NAFLD and no obesity	35 (30%)

Basic characteristics of disease parameters

A baseline assessment of disease parameters was performed for all subjects. The means and standard deviation (SD) values were calculated among the two groups. Weight differed significantly among groups (p<0.05). Similarly, systolic blood pressure (SBP), diastolic blood pressure (DBP), and C-reactive protein (CRP) were higher among the NAFLD and obesity group patients (p<0.05). The opposite was found with aspartate aminotransferase (AST), alanine aminotransferase (ALT), and gamma-glutamyl transferase (GGT), as it was higher in the NAFLD and no obesity group (p<0.05). There was no significant statistical difference between the groups for the rest of the parameters. Table 2 shows the basic characteristics of disease parameters of study subjects.

**Table 2:** Basic characteristics of disease parameters of study subjects at baseline **NA - not available due to missing data

Parameters	NAFLD and obesity (n= 81)	NAFLD and no obesity (n = 35)	p-value
	Mean ± SD	Mean ± SD	
Weight (kg)	82.9 ± 30.2	39.9 ± 19.8	<0.001*
Systolic blood pressure (SBP) mmHg	123.9 ± 15.2	112.0 ± 10.9	0.001*
Diastolic blood pressure (DBP) mmHg	69.9 ± 11.4	63.7 ± 14.6	0.034*
Triglycerides (TG) mmol/L	1.55 ± 1.48	1.55 ± 0.89	0.90
Total cholesterol (TC) mmol/L	4.43 ± 1.49	3.81 ± 1.27	0.10
High-density lipoprotein (HDL) mmol/L	3.09 ± 15.86	1.41 ± 1.46	0.67
Low-density lipoprotein (LDL) mmol/L	2.85 ± 1.20	2.31 ± 0.93	0.09
Insulin (mIU/L)	28.57 ± 18.34	NA**	NA**
Fasting blood glucose (FBG) mmol/L	6.95 ± 5.06	6.02 ± 3.55	0.97
Alanine aminotransferase (ALT) U/L	48.8 ± 58.6	49.2 ± 45.4	0.045*
Aspartate aminotransferase (AST) U/L	40.6 ± 52.7	65.0 ± 66.4	0.015*
Alkaline phosphatase (ALP) U/L	185.7 ± 85.8	238.8 ± 131.4	0.47
Gamma-glutamyl transferase (GGT) U/L	45.4 ± 101.6	64.6 ± 109.0	0.005*
Bilirubin umol/L	7.95 ± 4.67	15.91 ± 23.09	0.25
Prothrombin time (PT) seconds	14.81 ± 17.61	10.80 ± 2.49	0.44
C-reactive protein (CRP)	47.1 ± 112.5	1.21 ± 0.92	<0.001^*^

The radiology and liver biopsy histology reports of the subjects diagnosed with NAFLD were reviewed, and the diagnosis criteria were applied to determine the stage of NAFLD. After reviewing the medical records of the subjects, it was found that ultrasound was the most commonly used (n=110) radiological modality to diagnose NAFLD, followed by a CT scan (n=4) followed by liver biopsy (n=2). Simple steatosis was the most common stage of NAFLD among both groups (n=114), followed by moderate fatty liver and NASH (n=2 for both stages). None of the subjects had cirrhosis or fibrosis upon the first diagnosis. Table 3 shows the breakdown of the NAFLD diagnostic method and stages of NAFLD observed among the groups in the first assessments.

**Table 3:** NAFLD diagnosis and stages among groups NAFLD - non-alcoholic fatty liver disease

Variables	NAFLD and obesity (n=81), n (%)	NAFLD and no obesity (n=35), n (%)	Total (N=116)
1^st^ assessment NAFLD diagnosis method			
Ultrasound (US)	78 (71%)	32 (29%)	110
Computed tomography (CT)	3 (75%)	1 (25%)	4
Liver biopsy	0 (0%)	2 (100%)	2
1^st^ assessment NAFLD stage			
Mild simple fatty liver or steatosis	80 (71%)	32 (29%)	112
Moderate fatty liver or steatosis	1 (50%)	1 (50%)	2
NASH	0 (0%)	2 (100%)	2

Follow-up NAFLD diagnosis reports (radiology or histology) data were available for 37 patients. While 25 subjects were found to have simple steatosis, seven subjects were diagnosed with NASH, and three patients had normal liver. On the last follow-up, the ultrasound was still the highest modality for following up NAFLD (n=31), and six subjects underwent liver biopsy. Table 4 shows the NAFLD diagnostic method and stages in patients with a documented follow-up.

**Table 4:** Patients with a documented follow-up of NAFLD stages NAFLD - non-alcoholic fatty liver disease

Variables	NAFLD and obesity (n=81), n (%)	NAFLD and no obesity (n=35), n (%)	Total (N=116)
Follow-up assessment modality			
Ultrasound (US)	17 (55%)	14 (45%)	31
Computed tomography (CT)	0 (0%)	0 (0%)	0
Liver biopsy	2 (33%)	4 (67%)	6
Follow-up assessment of NAFLD stage			
Simple fatty liver or steatosis	14 (57%)	11 (43%)	25
Moderate fatty liver or steatosis	1 (50%)	1 (50%)	2
Non-alcoholic steatohepatitis (NASH)	2 (29%)	5 (71%)	7
Normal	2 (67%)	1 (33%)	3

## Discussion

This study showed that obese patients have a higher chance of having NAFALD and more severe NAFLD stages. Furthermore, data showed that patients with obesity combined with NAFLD have a more significant proportion, with more than two-thirds of patients being obese compared to non-obese. Thus, BMI could be considered a risk factor for NAFLD [21-23]. This study also showed that children older than ten are more likely to be diagnosed with NAFLD. Furthermore, results from different studies have concluded the same [9]. Furthermore, patients under 20 years old have a higher chance of being diagnosed with NAFLD by 20% [9, 21, 22]. Statistical analysis for basic characteristics of disease parameters of study subjects has shown that all the significant parameters are higher among NAFLD with obesity rather than those who are not obese, such as blood pressure, weight, etc. On the other hand, AST was the only parameter higher among NAFLD and non-obese patients than the obese group. The results for a higher range of AST among non-obese NAFLD patients might be attributed to a non-diagnosed medical condition other than NAFLD [9, 24].

According to Patton et al., Severe liver steatosis, moderate and mild, was 48%, 32%, and 20% of children have been diagnosed, respectively. On the other hand, the results of this study have shown that 71% of patients were diagnosed with mild severity. Furthermore, five out of the 37 patients who were followed up developed NASH [5]. There are limited studies on the progression of NAFLD in the pediatric population. However, it has been reported that up to 26% of patients with NAFLD could progress to advanced disease with liver fibrosis [25-26]. Also, fewer studies on NASH severity exist, especially in Asia [24]. Similar to other studies, the severity of NAFLD could be diagnosed through ultrasound, as most of the study subjects have been diagnosed (N=131) due to its ease and non-invasiveness. Also, it was diagnosed by CT scan in four patients and through liver biopsy in eight subjects [5, 21, 27].

This study is the first of its type among the Saudi population children, which gives it strength as it is the baseline for any further investigations. As obesity is associated with NAFLD and other comorbidities, lifestyle changes and behavioral, dietary, and physical activities are needed nationally. This study's limitations include the relatively small sample size and being retrospective. Moreover, all subjects have been diagnosed in the central region of Saudi Arabia, which might not represent the whole spectrum of the Saudi population. Finally, a few subjects have been followed, but there was insufficient data for further knowledge regarding NAFLD progression.

## Conclusions

The non-alcoholic fatty liver disease proportion is more significant among obese subjects rather than non-obese. NAFLD is commonly mild among children and adolescent Saudi patients. Ultrasound was the most common modality used in clinical practice for NAFLD diagnosis and follow-up in the study population. Further studies are needed to be conducted on NAFLD among the Saudi population to understand its prevalence, risk factors, and progression. Also, further investigations regarding the progression of different parameters, such as liver enzyme characterization over time, are needed to determine the comparison among different regions in Saudi Arabia.
